# Dr. Cayley, on Medical Reform

**Published:** 1806-10

**Authors:** George Cayley


### To Dr. HARRISON.

Sir,

*Ripon, June* 25, 1806.

THAT I have long lamented the degraded state of public opinion, and the fallen dignity of our profession, I beg beg to refer you for proof to vol. i. p. 404 of the Medical and Physical Journal. What I have there hinted will now, I hope, by your zeal and the very powerful influence you have obtained, be carried into effect; and I most sincerely hope that you will live to see so grand a scheme accomplished, and to receive that reward which surpasseth all others, which arises from a mind conscious of its own rectitude; and when the object accomplished is public good, the reward is great, and cannot be taken from you.

I am happy to find that many elevated and powerful persons are such strong advocates for the measure; to oppose it would be blindness: I have conversed with every one in this district, both in and out of the profession, except vain pretenders to medicine, and all decidedly approve of the measure.

The general outline of your plan agrees, I think, much with mine, and I think it very right that we should have the protection of the law in recovering our fees.

In admitting apprentices, great care should be taken to exclude persons of such low descriptions as we often see in the profession. To effect this, the Indenture should be upon a stamp of higher value, and the subsequent licence to practise, after a due examination, should also be beyond the reach of the dregs of society; I will not, however, enlarge upon the plan until I receive it in its more mature state; I will then, agreeably to your wish, give my opinion on each article.

I shall here subjoin a list of the names of those gentlemen in this town and neighbourhood, who obligingly attended a meeting I called for the purpose in the month of March last, and subscribed their names as approving of the measure; there are but two or three omitted.

I feel proud in offering this mite towards so laudable an undertaking, for which the original movers deserve the thanks of the nation.

I am, &c.

GEORGE CAYLEY, M. D.

---

WE, the undersigned, Inhabitants of Ripon and its vicinity, assembled at a meeting at the Unicorn Inn, in consequence of a Requisition signed by George Cayley, Doctor of Physic, to take into consideration the subject of a circular letter, addressed to the medical gentlemen, and issued from the Treasury, stating, that application is to be made to Parliament for a Medical Reform in this Kingdom;

Kingdom; with the intent that the Practice of Medicine shall in future be placed in the hands of respectable men, properly educated for the Profession, who shall undergo a regular examination before they shall obtain a licence to practise, whether Physicians, Surgeons, Apothecaries, or Druggists, after having been educated in such manner as the law shall direct; and as we find that it is a desirable object to give additional weight to the intended application, by taking the sense of gentlemen out of the profession upon this subject, we do hereby give it as our opinion that such a measure is highly necessary, and fraught with the greatest advantage, both as regarding the dignity and importance of the profession, and the welfare and security of our fellow citizens, accordingly do recommend it to the serious consideration of Parliament.

Then follow the names of nine professional gentlemen in Ripon, and upwards of forty out of the profession.

